# Expression profile analysis of two antisense lncRNAs to improve prognosis prediction of colorectal adenocarcinoma

**DOI:** 10.1186/s12935-019-1000-1

**Published:** 2019-11-06

**Authors:** Milad Shademan, Azam Naseri Salanghuch, Khadijeh Zare, Morteza Zahedi, Mohammad Ali Foroughi, Kambiz Akhavan Rezayat, Hooman Mosannen Mozaffari, Kamran Ghaffarzadegan, Ladan Goshayeshi, Hesam Dehghani

**Affiliations:** 1 Department of Basic Sciences, Faculty of Veterinary Medicine, Ferdowsi University of Mashhad, Azadi Square, Mashhad, 9177948974 Iran; 2 Division of Biotechnology, Faculty of Veterinary Medicine, Ferdowsi University of Mashhad, Mashhad, Iran; 3 Department of Gastroenterology and Hepatology, Faculty of Medicine, Mashhad University of Medical Sciences, Mashhad, Iran; 4 Surgical Oncology Research Center, Mashhad University of Medical Sciences, Mashhad, Iran; 5 Gastroenterology and Hepatology Research Center, Mashhad University of Medical Sciences, Mashhad, Iran; 6 Pathology Department, Education and Research Department, Razavi Hospital, Mashhad, Iran; 7 Stem Cell Biology and Regenerative Medicine Research Group, Research Institute of Biotechnology, Ferdowsi University of Mashhad, Mashhad, Iran

**Keywords:** Natural antisense transcripts, Colorectal adenoma, Colorectal adenocarcinoma, Transcription, PRKCQ-AS1, SATB1-AS1

## Abstract

**Background:**

Long noncoding RNAs (lncRNAs) are involved in different pathogenesis pathways including cancer pathogenesis. The adenoma-carcinoma pathway in colorectal cancer may involve the aberrant and variable gene expression of regulatory RNAs. This study was conducted to analyse the expression and prognosis prediction ability of two natural antisense transcripts, protein kinase C theta antisense RNA 1 (PRKCQ-AS1), and special AT-rich sequence binding protein 1 antisense RNA 1 (SATB1-AS1) in colorectal low-grade adenoma, advanced adenoma, and adenocarcinomas.

**Methods:**

In this study, from two RNA-seq analyses of CCAT1-ko cells and colorectal carcinoma biopsies having diminished and increased levels of CCAT1 transcription, respectively, we nominated two antisense lncRNAs of PRKCQ-AS1 and SATB1-AS1. Samples from colorectal low-grade adenomas, advanced adenomas, adenocarcinomas, and adjacent tissue were subjected to RT-qPCR to determine the expression of PRKCQ-AS1, SATB1-AS1 along with colon cancer-associated transcript 1 (CCAT1) and cMYC. In addition, we used different bioinformatics analyses and webservers (including GEPIA 2, TCGA, and CancerMine) to elucidate the prognosis prediction value, the expression correlation of sense–antisense pair of genes, and the expression profile of these antisense transcripts at the presence or absence of mutations in the driver genes, or the corresponding sense genes.

**Results:**

PRKCQ-AS1 showed a wide range of expression levels in colorectal adenoma, advanced adenoma, and adenocarcinoma. Upregulation of PRKCQ-AS1 was related to a significant decrease in survival of colorectal cancer (CRC) patients. The expression levels of PRKCQ-AS1 and PRKCQ were strong and significantly concordant in normal and cancerous colorectal tissues. While SATB1-AS1 showed a wide range of expression in colorectal adenoma, advanced adenoma, and adenocarcinoma as well, its expression was not related to a decrease in survival of CRC patients. The expression levels of SATB1-AS1 and SATB1 (the sense gene) were not strong in normal colorectal tissues. In addition, where SATB1 gene was mutated, the expression of SATB1-AS1 was significantly downregulated.

**Conclusions:**

We found the expression of PRKCQ-AS1 and SATB1-AS1 at a given stage of CRC very variable, and not all biopsy samples showed the increased expression of these antisense transcripts. PRKCQ-AS1 in contrast to SATB1-AS1 showed a significant prognostic value. Since a significantly concordant expression was observed for SATB1-AS1 and SATB1 in only cancerous, and for PRKCQ-AS1 and PRKCQ in both normal and cancerous colorectal tissues, it can be concluded that common mechanisms may regulate the expression of these sense and antisense genes.

## Background

Long non-coding RNAs (lncRNAs) play major roles in the regulation of chromatin architecture, transcription, nuclear domains, and nuclear bodies [1]. LncRNAs are involved in epigenetic regulation, and in response to environmental cues can adopt different structures and interact with different partners to organize chromatin organization and transcription. Involvement of lncRNAs in cellular phenomena of genome imprinting, dosage compensation, pluripotency, differentiation commitment, tissue-specificity of gene expression, and their aberrant functionality in different diseases underscores their importance in different pathogenesis pathways including cancer pathogenesis [2, 3].

LncRNAs are classified into three categories of mRNA-like intergenic transcripts (lincRNAs), antisense transcripts, and RNA polymerase II derived unconventional lncRNAs [1]. Natural antisense transcripts (NATs) are transcribed from the opposite strands of either protein-coding or noncoding genes, and are believed to make R-loops and tether the chromatin modifiers and remodelers to the locus, in order to regulate the genes in the opposite strand [1, 4]. Aside from the regulation of opposite genes, these antisense transcripts are also known to be involved in microRNA sponging.

The adenoma-carcinoma pathway accounts for approximately 75% of colorectal cancers [2]. In this pathway, the epigenetic deregulation of genome function, aberrant and variable gene expression, alteration of cell fate and identity, development of mutations in coding genes, cellular transformation, cellular heterogeneity, and other phenomena drive the cells to a pathologic state of adenocarcinoma [5]. Mounting evidence indicates the involvement of different categories of lncRNAs in cancer pathogenesis. For example, the colon cancer-associated transcript 1 (CCAT1) is an mRNA-like lincRNA that is upregulated in various human malignancies and is involved in different pathologic cellular processes of CRC [6–12]. And, the HOXA transcript antisense RNA, myeloid-specific 1 (HOTAIRM1) is a NAT that can directly regulate the spatial organization of chromatin and is involved in colorectal cancers [3, 13, 14].

Colorectal polyps are a combination of heterogeneous cells that can develop to multiclonal tumors, containing an indeterminate number of distinct clones of cells greater than one [15]. In fact, the relative frequency of different cell clones, affected by their environment and influenced by their own epigenome and genetic risk factors, may determine the progress of different population of cells (in polyps) to different carcinomas. Hence, identification of molecular players in the early non-advanced and advanced adenomas may prove to be very important for prognosis prediction and treatment strategies of advanced adenocarcinomas. In this study, we profile the expression of two NATs, protein kinase C theta antisense RNA 1 (PRKCQ-AS1), and special AT-rich sequence binding protein 1 antisense RNA 1 (SATB1-AS1) in colorectal low-grade adenomas, advanced adenomas, and adenocarcinomas. Furthermore using different bioinformatics analyses and webservers, we provide evidence and emphasize on the importance of PRKCQ-AS1 expression profiling for the pathogenesis and prognosis prediction of colorectal cancer.

## Methods

### Polyp and cancer biopsies

Before sampling, an informed consent questionnaire succinctly describing the research outline was described for each patient and was filled. Colorectal biopsy samples and polypectomy specimens were acquired from patients at the Gastroenterology Ward, Imam Reza Hospital, Mashhad University of Medical Sciences, Mashhad, Iran. The samples were immediately placed in pre-numbered cryovials containing RNA Shield (DENAzist Asia Co., Iran) and were transferred to liquid nitrogen within 30 min. Biopsy samples were also placed in containers with the same identification numbers and were sent to Mashhad Pathology Laboratory, Mashhad, for histopathological analysis. Samples from adjacent mucosa were prepared and processed as described above to be used as control. Frozen samples were transferred in portable containers of liquid nitrogen to Research Institute of Biotechnology, Ferdowsi University of Mashhad, and stored in − 80 freezer for further processing. A total of 12 low-grade adenoma, 14 advanced (high-grade) adenoma, and nine cancer tissue samples along with their adjacent mucosa were used in this study.

### Reverse transcription quantitative PCR

Total RNA was isolated from sample biopsies using Column RNA Isolation Kit (DENAzist Asia Co., Iran). The quality and quantity of extracted RNA were evaluated by gel electrophoresis and a Biotek Epoch 2 microplate spectrophotometer (Biotek, USA). Total RNA (5 μg) was reverse transcribed using random hexamer oligos and MMLV reverse transcriptase (Thermo Fisher Scientific, USA). Transcripts of five genes of CCAT1, cMYC, PRKCQ-AS1, SATB1-AS1, and beta-actin were quantified by specific dual-labeled hybridization probes (Table 1) in quantitative RT-PCR reactions containing Premix Ex Taq (Probe qPCR) master mix (Takara, Japan), cDNA from each sample, and specific primers, carried out in a Rotor-Gene Q real-time PCR cycler (Qiagen, USA).

**Table 1:** Oligonucleotides used in this study

Gene	Sequence (5′ to 3′)	Product (bp)	Application
CCAT1 (NR_108049.1)	F: CTGACAACATCGACTTTG R: CTCACAGTTTTCAAGGGA Probe: FAM-CTTAGCCATACAGAGCCAACCTG-BHQ1	108	qPCR
ACTB	F: TGCAGAAGGAGATCACTG R: CTTGCTGATCCACATCTG Probe: CY5-AAGATCAAGATCATTGCTCCTCCTGA-BHQ2	141	qPCR
MYC	F: TCCACAGAAACAACATCG R: CTCGGATTCTCTGCTCTC Probe: HEX-TTCTTCCTCATCTTCTTGTTCCTCCTC-BHQ1	147	qPCR
PRKCCQ-AS1 (NR_036501)	F: ACTGCTTTCAACTTTACTG R: AGTCCTCAGCATTATTCC Probe: FAM-AACCATCTTCTAGGCACAGTAGC-BHQ1	138	qPCR
SATB1-AS1 (NR_125803.1)	F: AAGGGTGGAAGAGTAAAC R: GTTGGATGAGAAAGTTCAG Probe: FAM-CCATCTTGACAGGAAGCAGAAGTTC-BHQ1	188	qPCR

QPCR reactions were repeated to adjust the reaction temperature, the concentration of primers, and to acquire the best amplification curves (Additional file 1: Figure S1). The identity of PCR products was confirmed by Sanger sequencing (Macrogen Inc., South Korea). Amplified fragments were sub-cloned in pTZ57R or pGATA plasmids, or extracted from gels, and their serial dilution (indicating a specific copy number) was used to make standard curves. Each dilution was subjected to three PCR reactions, and real-time readings were performed in triplicate. Then, the log10 of copy numbers for each gene was plotted against the cycle threshold (Ct) numbers to make a standard curve (Additional file 2: Figure S2). The slope of standard curves was used in the following equation: E = (10^−1/slope^ − 1) × 100% to calculate reaction efficiency. All standard curves were linear in the analyzed range with an acceptable correlation coefficient (R^2^) (Additional file 2: Figure S2). Gene expression ratios for each of the four genes of CCAT1, cMYC, PRKCQ-AS1, and SATB1-AS1 over beta-actin were calculated at each stage of low-grade adenoma, advanced adenoma, and adenocarcinoma using Pfaffl method of relative quantification [16]. The statistical differences between the expression ratios in different stages were analyzed by Mann–Whitney *U* test.

### Analyses of differential expression

Differentially expressed genes in our two datasets of CCAT1-ko versus CCAT1-wt HT-29 cells, and CRC cancer biopsy versus adjacent mucosa have been shown in volcano plots. Also, we used different bioinformatics tools and databases, including GEPIA 2 [17], a webserver that extracts data from the Cancer Genome Atlas (TCGA; https://portal.gdc.cancer.gov/) data portal and the GTEx database of normal tissues, to show differential expression of genes in adenocarcinoma samples.

### Analysis of survival-related genes

Overall survival analysis was performed on the basis of gene expression. For the test of the hypothesis, the log-rank test is used in GEPIA 2. The hazards ratio (HR) was selected based on the Cox PH model and the 95% confidence interval (CI) was selected to be displayed as the dotted line. The gene expression threshold of 50% (median value) was determined to split the high-expression and low-expression cohorts.

### Expression correlation analysis

Correlation analysis between sense and antisense genes was performed by pair-wise gene expression correlation analysis with the expression data of TCGA and GTEx, using the method of the Pearson correlation coefficient using the GEPIA 2 webserver. As previously suggested [18], we considered the following correlation coefficients: 0.00–0.19 as very weak, 0.20–0.39 as weak, 0.40–0.59 as moderate, 0.60–0.79 as strong, and 0.80–1.0 as very strong.

### Analysis of gene expression in relation to mutations in driver genes

Six driver genes were selected in colon cancer using CancerMine webserver [19]. Based on the number of citations in CancerMine, these six driver genes were APC, BRAF, TP53, PIK3CA, SMAD4, and EGFR. In TCGA portal 361 cases with mutations in these driver genes versus 11 cases without any mutation in these genes were selected and the expression profiling files were extracted in order to analyze the expression levels of PRKCQ-AS1 and SATB1-AS1 and to compare using *t*-test.

## Results

### Expression profiling of anti-sense lncRNA of PRKCQ-AS1 shows a wide range of expression in colorectal adenoma, advanced adenoma, and adenocarcinoma

**Fig. 1:**
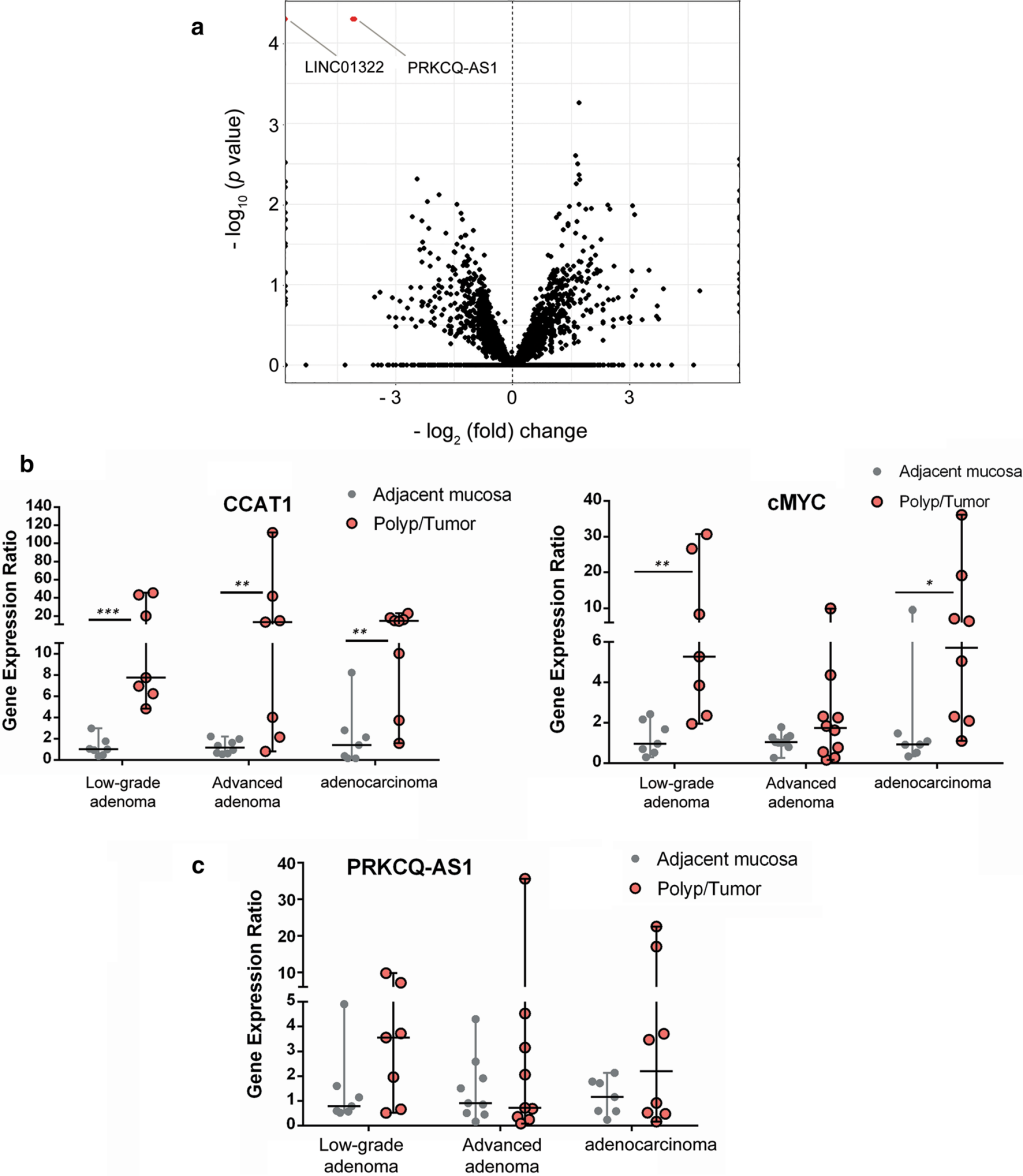
Volcano plot of differentially expressed genes in CCAT1-ko cells and expression profiling of CCAT1, cMYC and PRKCQ-AS1 in patient samples. **a** Volcano plot of differentially expressed genes (*p* < 0.001) in CCAT1-wt and CCAT1-ko HT-29 colorectal adenocarcinoma cells. The horizontal axis shows the log2 fold up-regulation (to the right) and down-regulation (to the left) in the expression of genes between CCAT1-wt and CCAT1-ko HT-29 cells. The vertical axis demonstrates negative log10 of the *p*-value of Fisher’s exact test. Each gene is represented by one circle on the graph. Red circles represent genes that are significantly de-regulated. **b** Quantified expression of CCAT1 (left panel) and cMYC (right panel) in the polyp/tumoral tissue in comparison with normal tissue in patients with low-grade adenoma, advanced adenoma, and adenocarcinoma. **c** Expression profiling of PRKCQ-AS1 in the polyp/tumoral tissue in comparison with normal tissue in patients with low-grade adenoma, advanced adenoma, and adenocarcinoma. Relative expression levels were determined by Pfaffl analysis, using beta-actin as a reference gene. The statistical analysis between the level of expression of each polyp/tumoral tissue and its adjacent normal mucosa was performed with Mann–Whitney test at significance levels of *p* < 0.05 (*), *p* < 0.01 (**), *p* < 0.001 (***)

CCAT1 is a lincRNA that is involved in different pathologic cellular processes of CRC. To identify non-coding RNAs that could be associated and regulated with CCAT1, CCAT1-knockout and CCAT1-wild type cells [20] were subjected to RNA-seq analysis. This analysis displayed a statistically significant differential expression of four long non-coding RNA genes (Fig. 1a). In CCAT1-knockout cells, the level of PRKCQ-AS1 significantly (*p* < 0.001) decreased more than 23 folds, indicating a possible regulation (direct or indirect) by CCAT1 (Fig. 1a). These findings prompted us to investigate (using RT-qPCR) whether this anti-sense transcript could also be activated along with CCAT1 in different stages of colorectal adenoma to adenocarcinoma. CCAT1 transcript levels were significantly increased in all stages of low-grade adenoma, advanced adenoma, and adenocarcinoma compared to the adjacent tissue (Fig. 1b). Also cMYC, a gene that its expression has been correlated to CCAT1 expression and activity, showed very similar expression profile (Fig. 1b). However PRKCQ-AS1, while showing a wide range of transcription (well below and above the median), it was not significantly upregulated in any of the three stages of colorectal adenoma, advanced adenoma, and adenocarcinoma (Fig. 1c).

**Fig. 2:**
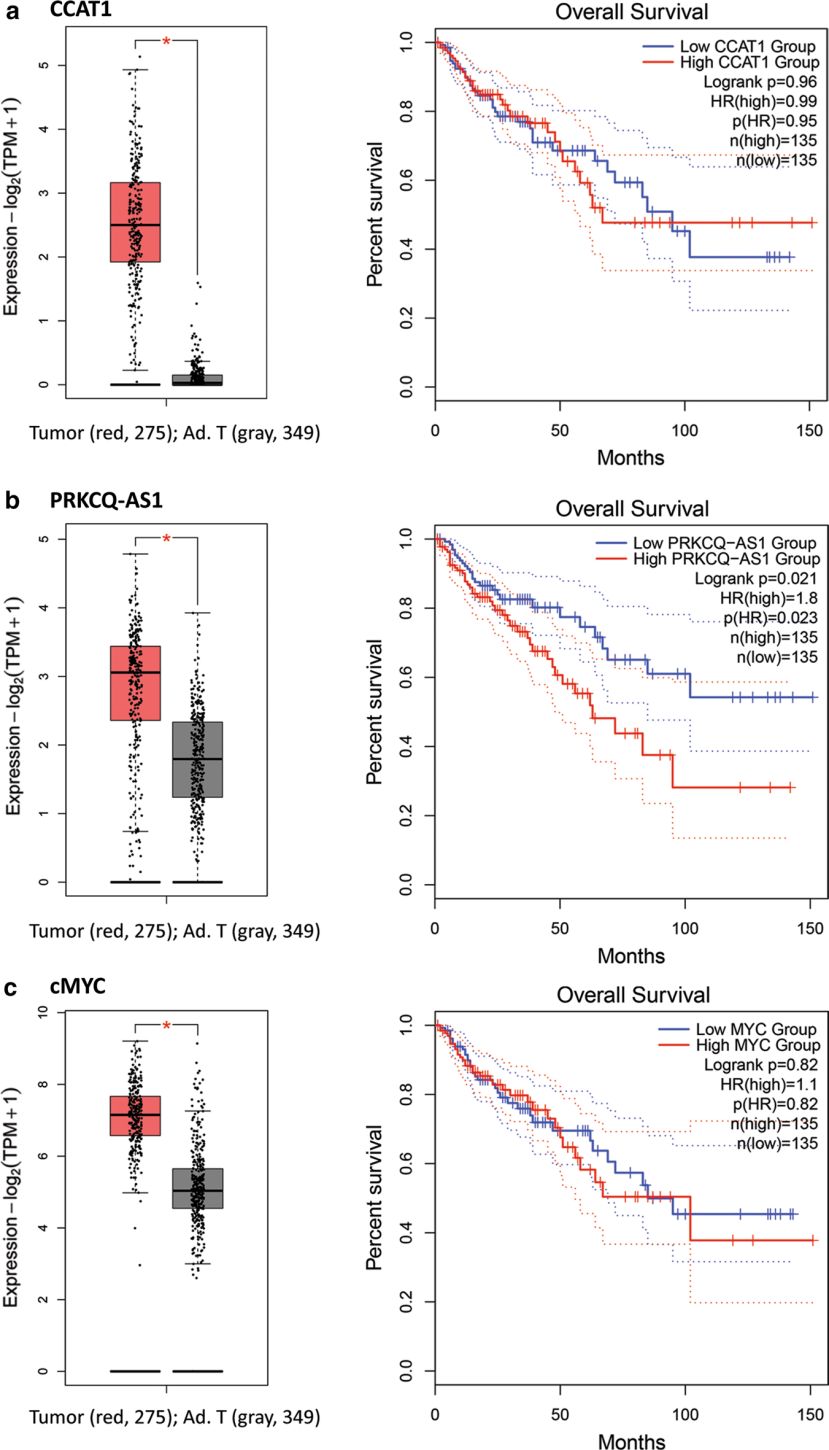
The expression profiling, and survival analysis of expression in TCGA colorectal adenocarcinoma patients. **a** The expression profiling of CCAT1, and correlation of overall survival to CCAT1 expression in colorectal cancer. **b** The expression profiling of PRKCQ-AS1 and correlation of overall survival to PRKCQ-AS1 expression in colorectal cancer. **c** The expression profiling of cMYC, and correlation of overall survival to cMYC expression in colorectal cancer

Then, we looked at the transcript levels of CCAT1, PRKCQ-AS1, and cMYC in TCGA cohort of 275 CRC and 349 normal tissues (Fig. 2a, b). A wide range of PRKCQ-AS1 expression levels above and below the median was also observed in RNA-seq analysis of TCGA data, confirming our RT-qPCR analysis and indicating that the increased expression of this lncRNA does not occur in every individual sample. This is in contrast to the observed profile of CCAT1 and cMYC that seem to be overexpressed in the majority of samples in any of the three stages of colorectal adenoma, advanced adenoma, and adenocarcinoma. These findings also suggest that in CRC cell line (where CCAT1 gene knockout has been performed) in comparison with colorectal tissues, PRKCQ-AS1 transcription might be regulated with different mechanisms. This speculation is supported in part by the analysis on TCGA datasets showing that the expression levels of PRKCQ-AS1 and CCAT1 have very weak correlation, while CCAT1 and cMYC demonstrate a strong correlation (Additional file 3: Figure S3).

### The anti-sense lncRNA of PRKCQ-AS1 demonstrates a significant potential for the prognosis prediction of overall survival in colorectal adenocarcinoma

Profiling the expression of this anti-sense RNA and study of its correlation with survival in TCGA CRC data (via GEPIA 2 webserver) [17], revealed that this gene has a significant prognostic value and its upregulation is related to a significant (p = 0.021) decrease in survival of CRC patients (Fig. 2b). This is in sharp contrast to the states of CCAT1 and cMYC upregulation, which their expression is not related to a significant decrease in survival of CRC patients (Fig. 2a, c). Hence, from these data, we could speculate that not all CRC tissues may show PRKCQ-AS1 upregulation, and those CRC patients with high levels of PRKCQ-AS1 may have less chance of survival.

### The expression level of PRKCQ-AS1 is strongly correlated with the expression of its sense gene, but it is not affected by mutation of driver genes

**Fig. 3:**
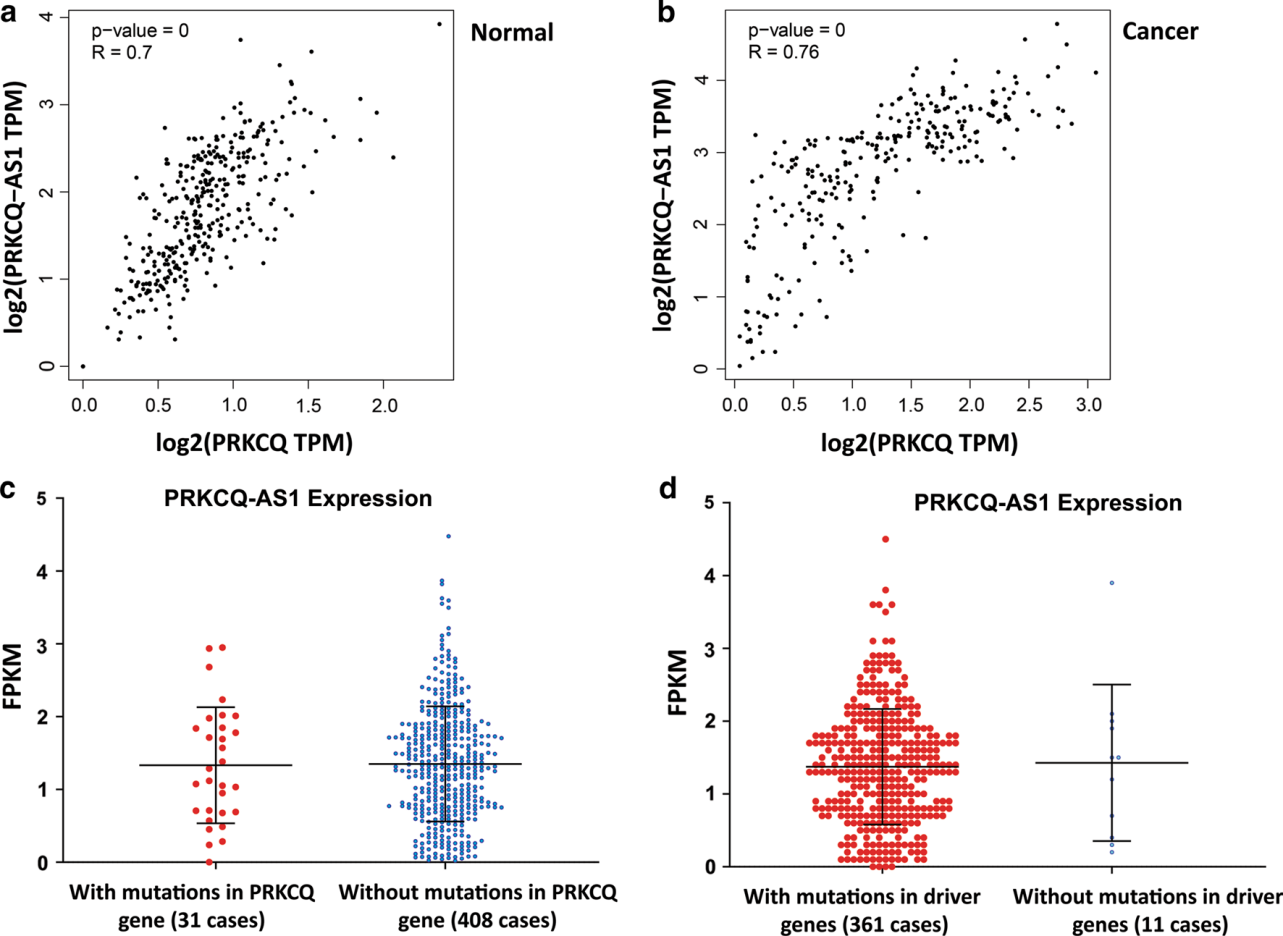
The expression profiling of PRKCQ-AS1 in normal and cancer tissues and in relation to mutations in driver genes and PRKCQ gene. **a** The strong correlation of expression of PRKCQ-AS1 and PRKCQ in normal colorectal samples. **b** Strong correlation of expression of PRKCQ-AS1 and PRKCQ in colorectal cancer samples. **c** Expression of PRKCQ-AS1 (as FPKM) at the presence or the absence of mutations in the PRKCQ gene. **d** Expression of PRKCQ-AS1 (as FPKM) at the presence or the absence of mutations in driver genes. Driver genes including APC, BRAF, TP53, PIK3CA, SMAD4, and EGFR were selected from CancerMine [19] for colorectal cancer

As it has been previously reported for the majority of NATs, an overall positive correlation exists between the expression of sense and antisense genes [4]. Our analysis on TCGA datasets revealed that the expression levels of PRKCQ-AS1 and PRKCQ were strong and significantly concordant (same direction) in both normal and tumor states (Fig. 3a, b), suggesting that common regulatory mechanisms might be involved to regulate the transcription of both sense and antisense genes. The expression of the PRKCQ-AS1 gene was not affected by the mutated or intact state of its sense gene (PRKCQ), suggesting that the expression of PRKCQ-AS1 is not likely to be regulated by the PRKCQ transcript (Fig. 3c). Our analysis on 361 TCGA CRC versus 11 normal cases showed that the expression of PRKCQ-AS1 was not affected where major driver genes (APC, BRAF, TP53, PIK3CA, SMAD4, and EGFR) were either intact or mutated (Fig. 3d). One of the implications for this finding could be that the expression of PRKCQ-AS1 in colorectal adenocarcinoma might not be under the control of major driver genes or the pathologic network of genes involving these drivers.

### SATB1-As1 is deregulated in colorectal cancer, but its expression is not highly correlated with poor survival

**Fig. 4:**
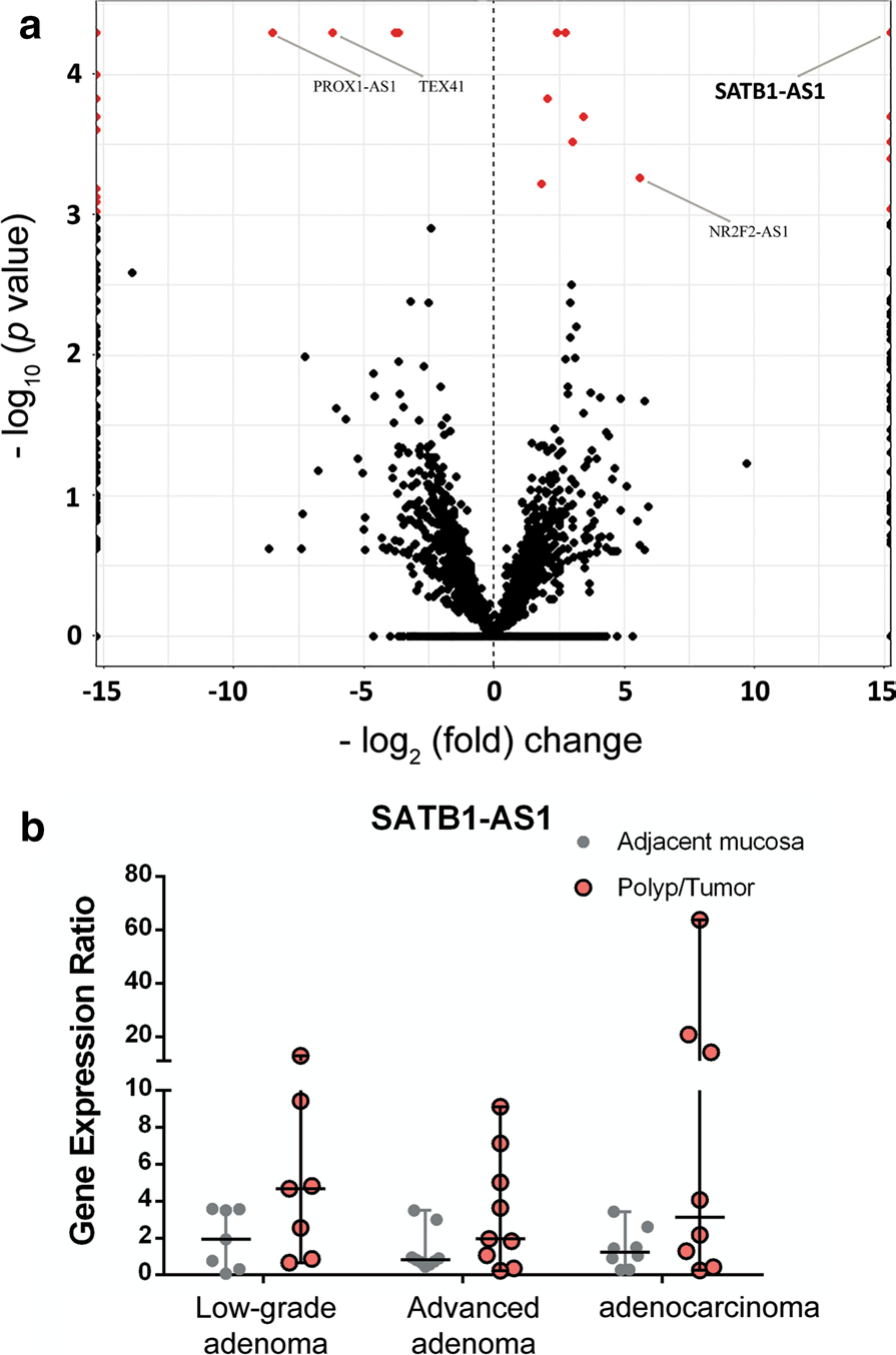
Volcano plot of differentially expressed genes in CRC and expression profiling of SATB1-AS1 in patient samples. **a** Volcano plot of differentially expressed genes (*p* < 0.001) in tumoral versus adjacent mucosal colorectal adenocarcinoma samples. The horizontal axis shows the log2 fold up-regulation (to the right) and down-regulation (to the left) in the expression of genes. The vertical axis demonstrates negative log10 of the *p*-value of Fisher’s exact test. Each gene is represented by one circle on the graph. Red circles represent genes that are significantly de-regulated. **b** Quantified expression of SATB1-As1 in the polyp/tumoral tissue in comparison with normal tissue in patients with low-grade adenoma, advanced adenoma, and adenocarcinoma

To identify non-coding RNAs that could be associated and regulated in a state with increased CCAT1 expression, we chose CRC tumoral tissue and its control adjacent tissue to perform RNA-seq analysis. The anti-sense lncRNA of SATB1-AS1 was significantly (*p* < 0.001; more than 215-fold) upregulated (Fig. 4a).

**Fig. 5:**
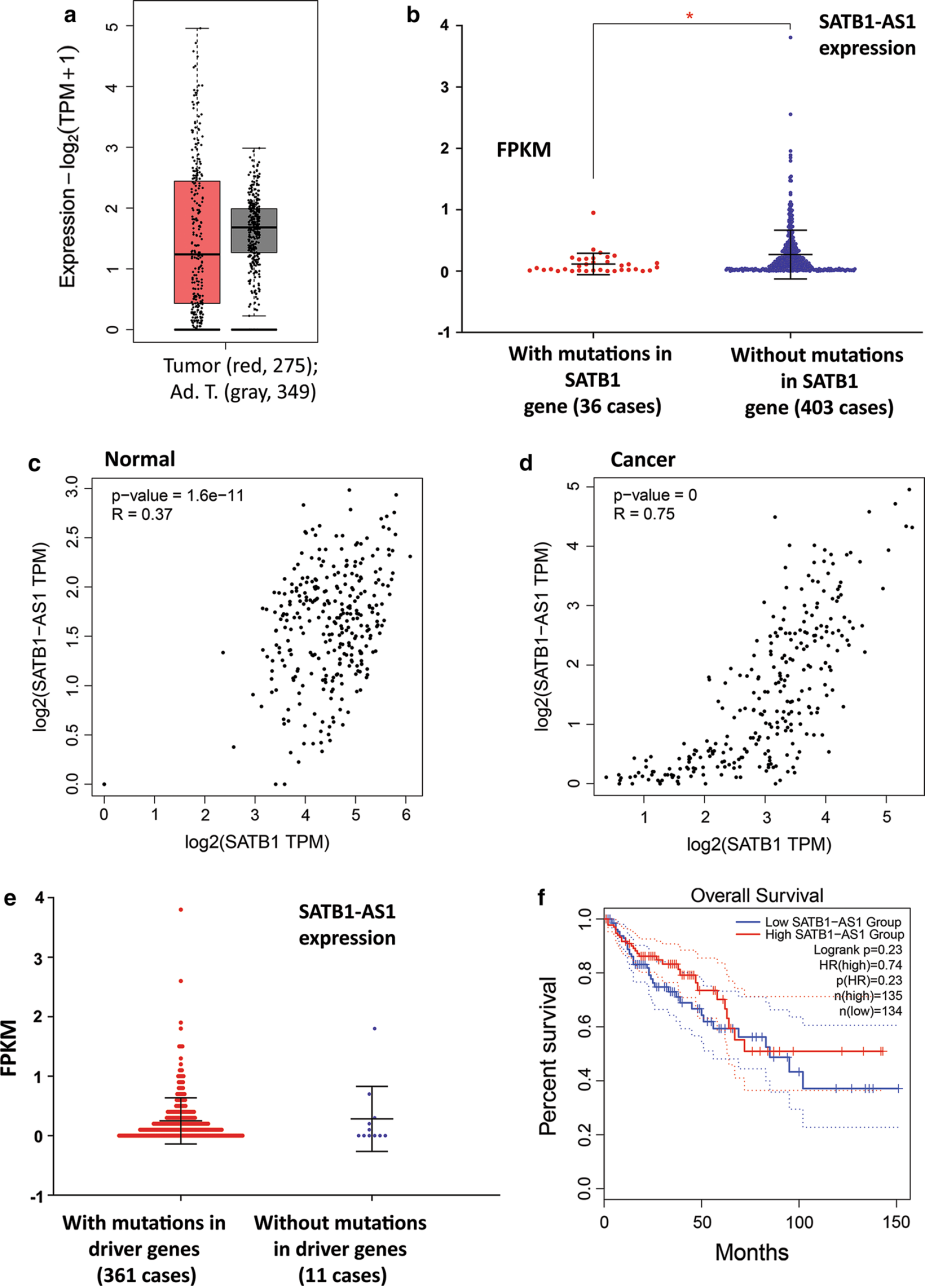
The expression profiling and survival analysis of expressions of SATB1-AS1 in TCGA colorectal adenocarcinoma patient samples. **a** The expression profiling of SATB1 in tumoral and normal tissues of TCGA colorectal adenocarcinoma samples. **b** Expression of SATB1-AS1 (as FPKM) at the presence or the absence of mutations in the SATB1 gene. **c** Weak correlation of expression of SATB1-AS1 and SATB1 in normal colorectal samples. **d** The strong correlation of expression of SATB1-AS1 and SATB1 in colorectal cancer samples. **e** Expression of SATB1-AS1 (as FPKM) at the presence or the absence of mutations in driver genes. **f** Correlation of overall survival to SATB1 expression in TCGA colorectal adenocarcinoma datasets

We used RT-qPCR to profile the expression of this antisense transcript in different stages of colorectal adenoma to adenocarcinoma. Similar to PRKCQ-AS1, while showing varying levels of transcription (well below or above median), SATB1-AS1 was not significantly up- or down-regulated in any of the three stages of colorectal adenoma, advanced adenoma, and adenocarcinoma (Fig. 4b). Accordingly, when we analyzed the expression levels of SATB1 in TCGA data sets (through GEPIA2 webserver), while expression levels were widely well below or above the median, significant deregulation was not observed (Fig. 5a).

The expression of SATB1-AS1 gene was down-regulated in colorectal adenocarcinoma patients with SATB1 gene mutation (36 mutant versus 403 normal cases), suggesting that SATB1 might regulate its anti-sense lncRNA (Fig. 5b). While the expression levels of SATB1-AS1 and SATB1 were not significantly correlated in the normal state (Fig. 5c), their expression was correlated in cancer state (Fig. 5d). A possible explanation could be that a common mechanism could regulate the expression of sense and antisense genes for this locus in the cancerous state, but not in a normal state. Analysis of 361 TCGA CRC and 11 normal cases showed that the expression of SATB1-AS1 is not affected where major driver genes are mutated (Fig. 5e). And finally, the study of the correlation of SATB1-AS1 expression with survival revealed that the down-regulation of this gene was not significantly related to a decrease in survival of of TCGA CRC patients (Fig. 5f).

## Discussion

In this study, we profile the expression of two antisense transcripts of PRKCQ-AS1 and SATB1-AS1, along with CCAT1, and cMyc in colorectal low-grade adenomas, advanced adenomas, and adenocarcinomas. In addition, by analyzing TCGA datasets using GEPIA 2 webserver, we analyze the differential signature score and prognostic impact of these genes. We also identified the correlation between the signature scores of PRKCQ-AS1 and SATB1-AS1 and signature scores of their sense genes, PRKCQ and SATB1, respectively. The overall findings suggest that PRKCQ-AS1 may have a prognostic value for CRC and early adenomatous stages.

Antisense transcripts can have prognostic value for many diseases. HOX transcript antisense RNA (HOTAIR) for example, is significantly overexpressed in multiple tumors including breast cancer [21], colorectal cancer [22], thyroid cancer [23], pancreatic cancer [24] and gastric cancer [25]. HOTAIR’s up-regulation is associated with poor prognosis. The over-expression of a lncRNA in cancer tissue is identified by analysis of total RNA of the biopsied sample. Thus, given the heterogeneous nature of colorectal cancer, detection of a higher level of a given lncRNA (including antisense RNA) transcript could be due to very high expression of the gene in some cells (and not all cells) of the tumor (and tumor biopsy). A significant correlation between upregulation of an antisense transcript and survival in a patient, therefore, could be related to clonal proliferation of high-expressing cells compared to other cells. Our findings show that while PRKCQ-AS1 demonstrates a high impact for prognosis prediction (Fig. 2b); it has different levels of expression (ranging from well above the median to well below median) in different patient samples (Figs. 1c and 2b). This can be attributed to the clonal proliferation of high-expressing cells in some patient samples. This explanation emphasizes the importance of knowing the molecular mechanisms of transcriptional control for a given lncRNA and identification of factors that affect its transcription. It is interesting to note that CCAT1 and cMYC that show significantly higher expression levels in colorectal low-grade adenomas, advanced adenomas, and adenocarcinomas, do not provide a significant prognosis prediction impact (Figs. 1 and 2). Thus, the expression level of a given gene may be high or low in the majority of patient samples, without a prognosis prediction impact. In contrast, the expression level of a gene may be widely diverged in different patient samples with a significant prognosis prediction impact.

In contrast to our findings in CCAT1 knockout cells, the transcription of PRKCQ-AS1 did not follow CCAT1 transcription in different stages of colorectal adenoma to adenocarcinoma. This can be explained by a possibility that in the cell line hosting the knockout gene (HT-29) in comparison with the colorectal tissue, different regulatory mechanisms may exist for CCAT1 and PRKCQ-AS1 transcription. Another explanation could be that while the lack of expression of CCAT1 in knockout cells may have a profound effect on a network of genes including PRKCQ-AS1, the zero level of expression of CCAT1 is never achieved and repeated in colorectal tissues. And thus, the increased transcription of PRKCQ-AS1 in cells with increased expression of CCAT1 should not be expected.

Since colorectal polyps and tumors are heterogeneous and multiclonal [15], and the Darwinian evolution of colorectal cancer starts from early adenomas [26], they are expected to show varying levels of expression for a specific gene. This justifies the very wide levels of expression of different genes in different biopsies; in our study more than 30 folds for PRKCQ-AS1 and more than 60 folds for SATB1-AS1 (Figs. 1 and 4). However, it is important to identify the role of the overexpressed antisense transcript during cancer pathogenesis. Different functions have been defined for antisense lncRNA transcripts including chromatin remodeling of the target gene, regulation of the alternative splicing of the sense transcript, miRNA sponging, and masking of miRNA binding site on sense RNA [27]. The antisense transcripts can either repress or promote the expression of their sense gene. In our analysis, we found that in CRC the expression of PRKCQ-AS1 and SATB1-AS1 is significantly correlated with the expression of PRKCQ and SATB1, respectively (Figs. 3 and 5). It has been postulated that significantly concordant (same direction) or discordant (opposite direction) expression levels for a pair of sense and antisense genes may indicate common mechanisms regulating the expression of two genes. However, a recent report shows that sense-antisense pairs whose expression is strongly—positively or negatively—correlated can be regulated independently [28]. This suggests the requirement for functional studies to reveal the mechanism of expression and the nature of concordant or discordant expression correlations. Further experiments will be required to identify the role of PRKCQ-AS1 and SATB1-AS1 in CRC pathogenesis and the importance of the level of expression of the sense-antisense pairs in colorectal cancer.

Our analysis of antisense gene expression in relation to mutations in sense genes and driver genes shows that the expression of PRKCQ-AS1 is not affected and deregulated when the sense gene (PRKCQ) or driver genes (APC, BRAF, TP53, PIK3CA, SMAD4, and EGFR) are mutated. By multi-region whole-exome sequencing, Mimori and colleagues [26] report that early tumors accumulate a higher proportion of subclonal driver mutations than the advanced tumors, and that the variant allele frequencies of subclonal mutations tend to be higher in early tumors. These authors suggest that the subclonal mutations are subject to selective sweep in early tumorigenesis. Again, the heterogeneous nature of cells in the early or advanced adenomas and adenocarcinomas may explain the lack of correlation (Fig. 3c, d). The expression of SATB1-AS1 however, is significantly decreased in CRC samples that comprise mutations in the sense gene of SATB1 (Fig. 5b). This could be an indication of the fact that SATB1 is involved in the regulation of transcription of SATB1-AS1. We did not find any changes in the expression of SATB1-AS1, where driver genes were mutated, indicating the heterogeneous nature of cells in CRC, and emphasizing on this point that individual cell analysis for the expression of lncRNAs and analysis of mutations might be necessary to perform.

## Conclusions

In conclusion, we have provided the expression profile analysis of two antisense transcripts of PRKCQ-AS1 and SATB1-AS1, CCAT1 lncRNA, and cMYC in colorectal low-grade adenomas, advanced adenomas, and adenocarcinomas. Although PRKCQ-AS1 expression is not high in all colorectal biopsies, it demonstrates a high impact for prognosis prediction of colorectal cancer and survival. In this report, we argue that in early stages of colorectal adenomas (even in low-grade non-advanced polyps), the expression of a gene and its ratio to a reference gene could have a prognostic impact, and this expression may not be affected by mutations in driver genes or mutations in the sense gene.

## Supplementary information

**Additional file 1: Figure S1.** Representative amplification curves and gel electrophoresis of the amplified products for ACTB, MYC, PRKCQ-AS1, CCAT1 and SATB1-AS1 genes. A) CCAT1 amplification curve, B) PRKCQ-AS1 amplification curve, C) SATB1-AS1 amplification curve, D) ACTB (beta-actin) amplification curve, and E) cMYC amplification curve. F) RT-qPCR amplified products separated on the 2% agarose gel. a) ACTB (beta-actin) 141 base pairs, b) DNA size marker (50 bp), c) cMYC (147 bp), d) Upper band: PRKCQ-AS1-001 (NR_036501; 138 bp), lower band: PRKCQ-AS1-004 (NR_036503; 74 bp), e) CCAT1 (108 bp), f) SATB1-AS1 (188 bp).

**Additional file 2: Figure S2.** Representative Standard curves of 5 genes. A) SATB1-AS1, B) CCAT1, C) ACTB (beta-actin), D) PRKCQ-AS1, and E) cMYC.

**Additional file 3: Figure S3.** Correlation of expression of CCAT1 with cMYC, PRKCQ-AS1, and SATB1-AS1 in colorectal cancer samples. A) The strong correlation of expression of CCAT1 and cMYC in colorectal cancer samples. B) The very weak correlation of expression of CCAT1 and PRKCQ-AS1 in colorectal cancer samples. C) The very weak correlation of expression of CCAT1 and SATB1-AS1 in colorectal cancer samples.

## Data Availability

The data sets used and/or analysed during the current study are available from the corresponding author on reasonable request.

## Abbreviations

lncRNAs: long noncoding RNAs
PRKCQ-AS1: protein kinase C theta antisense RNA 1
SATB1-AS1: special AT-rich sequence binding protein 1 antisense RNA 1
CRC: colorectal cancer
NATs: natural antisense transcripts
CCAT1: colon cancer-associated transcript 1
HOTAIR: HOX transcript antisense RNA
