# Supplementary figures and images for: Expression profile analysis of two antisense lncRNAs to improve prognosis prediction of colorectal adenocarcinoma
Source: Cancer Cell Int. 2019 Nov 6;19:278. doi: 10.1186/s12935-019-1000-1 (PMC6836367; doi:10.1186/s12935-019-1000-1)

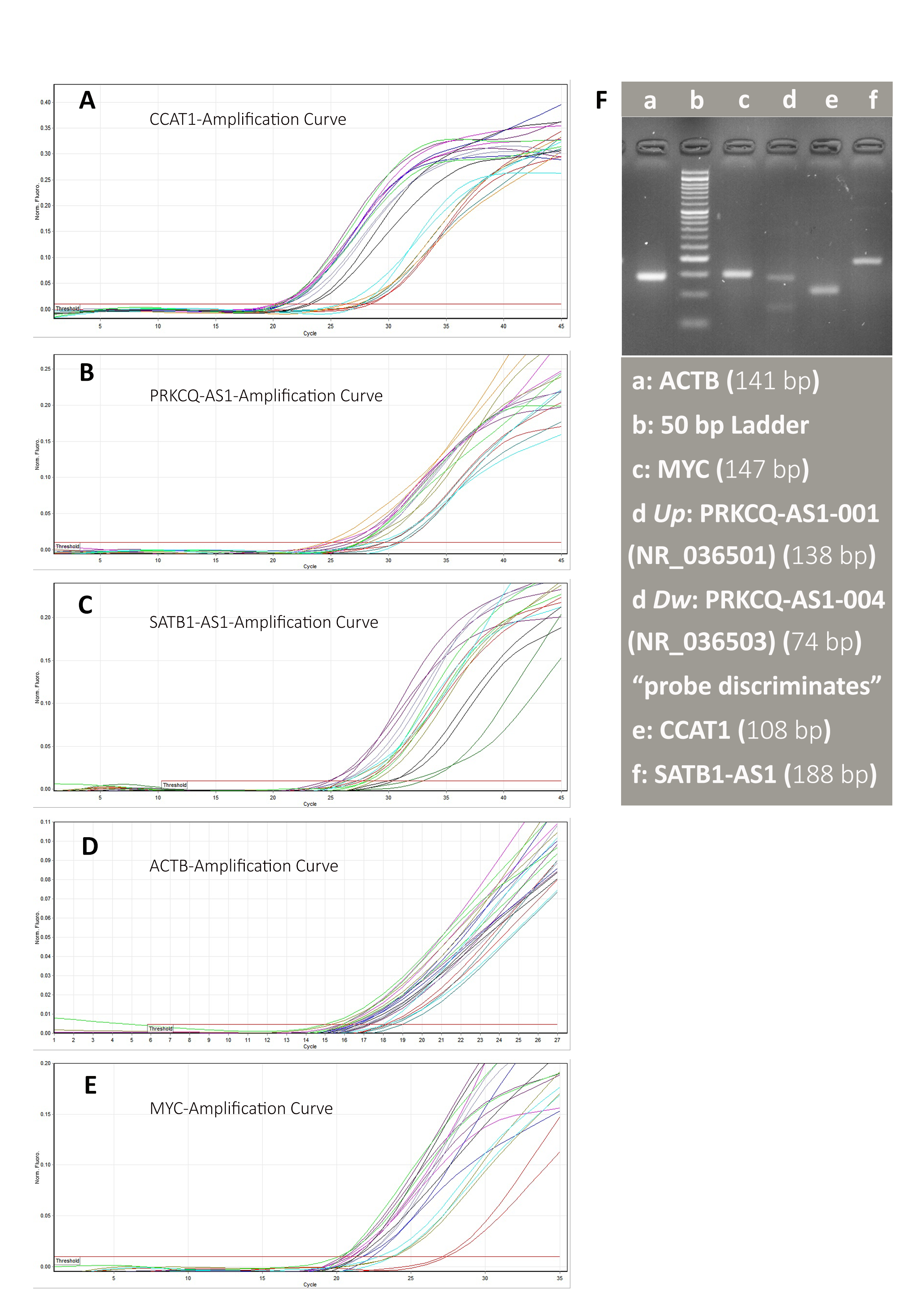

Supplement: Supplementary file 1 — Additional file 1: Figure S1. Representative amplification curves and gel electrophoresis of the amplified products for ACTB, MYC, PRKCQ-AS1, CCAT1 and SATB1-AS1 genes. A) CCAT1 amplification curve, B) PRKCQ-AS1 amplification curve, C) SATB1-AS1 amplification curve, D) ACTB (beta-actin) amplification curve, and E) cMYC amplification curve. F) RT-qPCR amplified products separated on the 2% agarose gel. a) ACTB (beta-actin) 141 base pairs, b) DNA size marker (50 bp), c) cMYC (147 bp), d) Upper band: PRKCQ-AS1-001 (NR_036501; 138 bp), lower band: PRKCQ-AS1-004 (NR_036503; 74 bp), e) CCAT1 (108 bp), f) SATB1-AS1 (188 bp). [file 12935_2019_1000_MOESM1_ESM.tif]

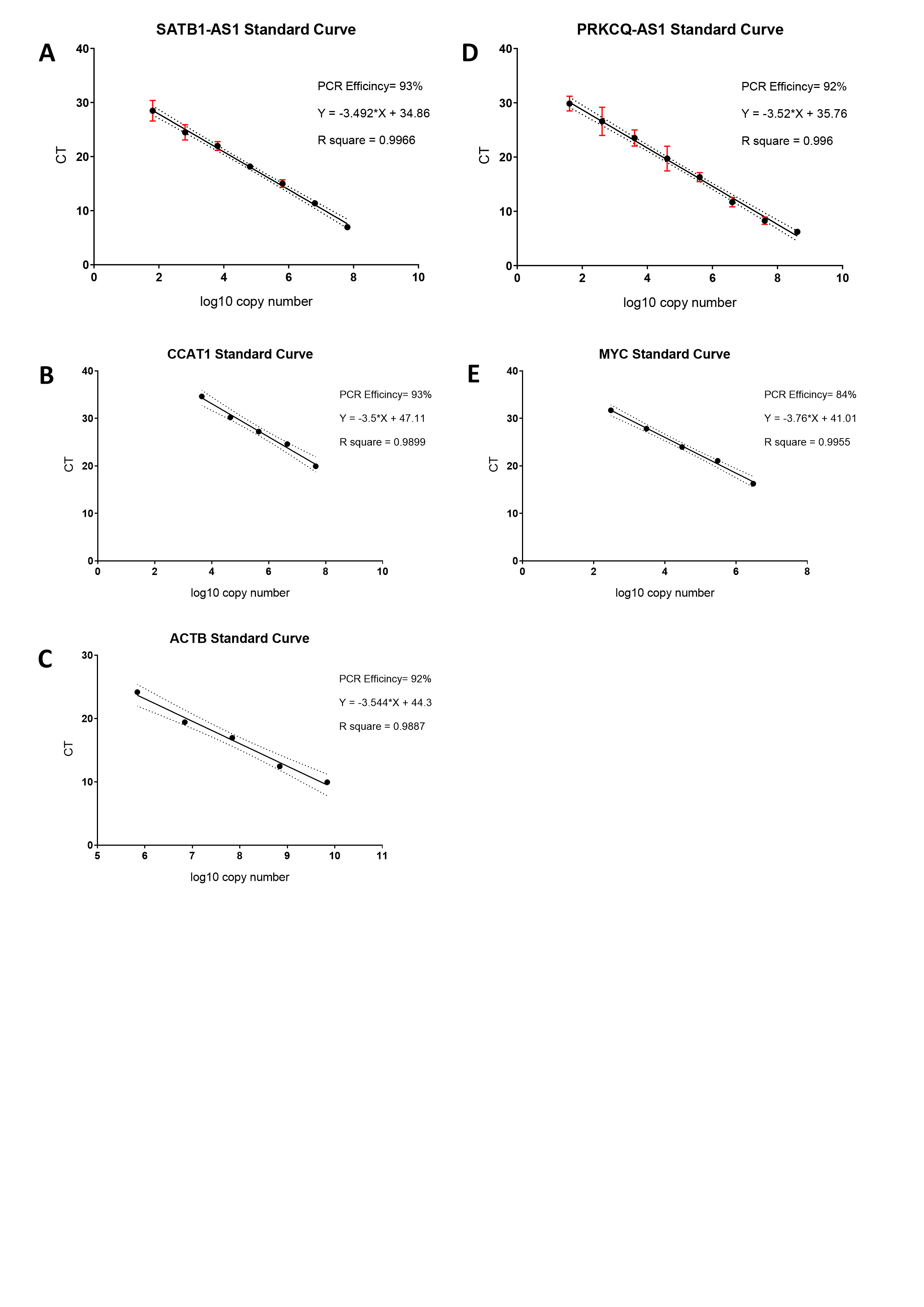

Supplement: Supplementary file 2 — Additional file 2: Figure S2. Representative Standard curves of 5 genes. A) SATB1-AS1, B) CCAT1, C) ACTB (beta-actin), D) PRKCQ-AS1, and E) cMYC. [file 12935_2019_1000_MOESM2_ESM.tif]

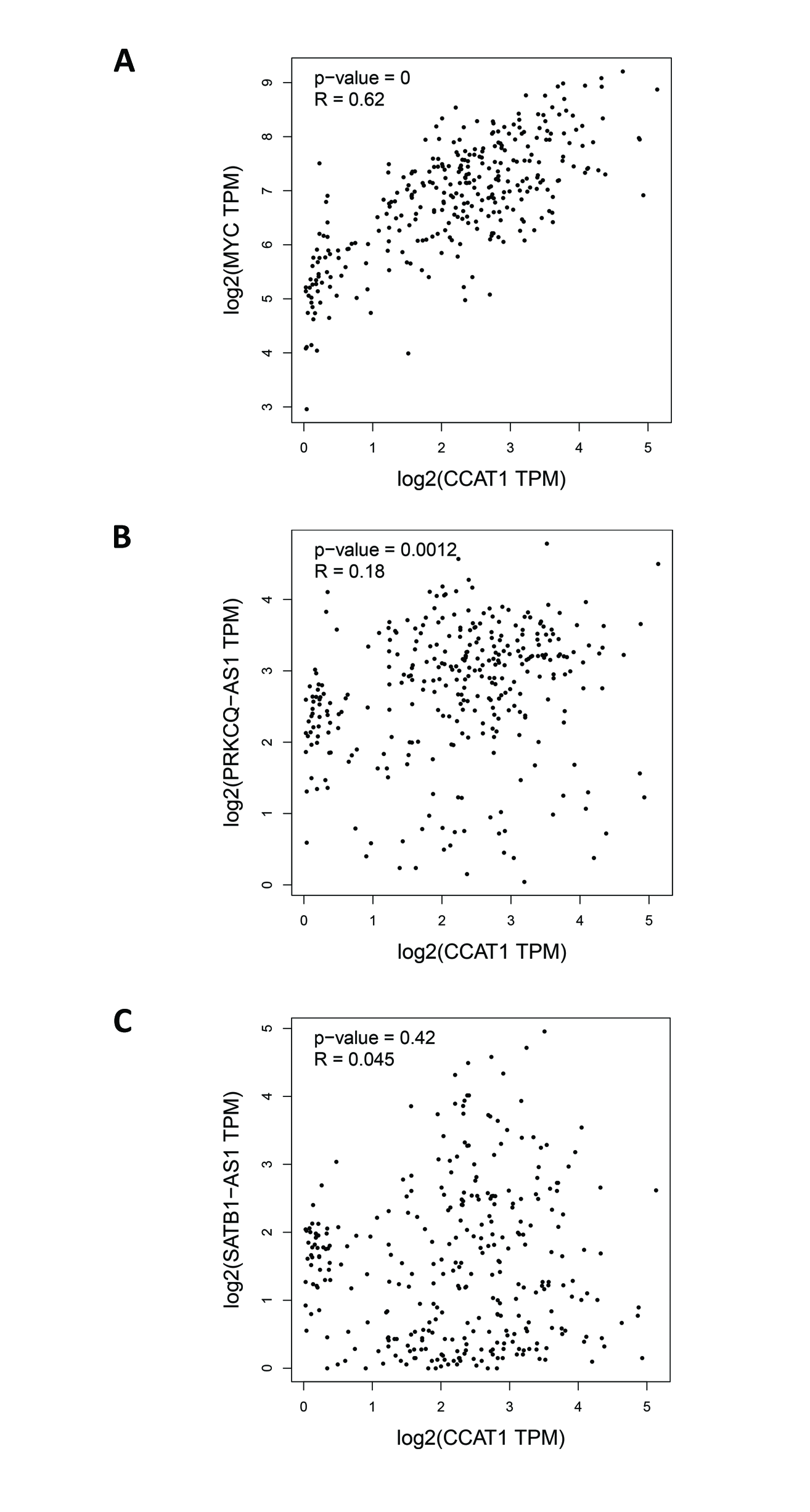

Supplement: Supplementary file 3 — Additional file 3: Figure S3. Correlation of expression of CCAT1 with cMYC, PRKCQ-AS1, and SATB1-AS1 in colorectal cancer samples. A) The strong correlation of expression of CCAT1 and cMYC in colorectal cancer samples. B) The very weak correlation of expression of CCAT1 and PRKCQ-AS1 in colorectal cancer samples. C) The very weak correlation of expression of CCAT1 and SATB1-AS1 in colorectal cancer samples. [file 12935_2019_1000_MOESM3_ESM.tif]
